# Success rate and duration of orotracheal intubation of premature infants by healthcare providers with different levels of experience using a video laryngoscope as compared to direct laryngoscopy in a simulation-based setting

**DOI:** 10.3389/fped.2022.1031847

**Published:** 2022-11-24

**Authors:** Nicolaus V. Salis-Soglio, Helmut Hummler, Stephan Schwarz, Marc R. Mendler

**Affiliations:** ^1^ Divison of Neonatology, Department of Pediatrics, University of Ulm, Ulm, Germany; ^2^ Divison of Neonatology, Department of Pediatrics, University of Tübingen, Tübingen, Germany

**Keywords:** video laryngoscopy, intubation, neonate, premature infant, intubation time, intubation attempt

## Abstract

**Background:**

Endotracheal intubation of very low birth weight infants (VLBWI) is an essential procedure in NICUs, but intubation experience is often limited. Video laryngoscopy (VL) has been described as a tool to improve intubation skills, but studies in high-risk neonatal populations are limited.

**Objective:**

The aim of this study was to investigate whether VL is a useful tool to support airway management in high-risk premature infants with inexperienced operators.

**Methods:**

In this crossover study predominantly inexperienced participants were exposed in random sequence to VL and conventional direct laryngoscopy (DL) for endotracheal intubation of a VLBWI simulation manikin to measure total time, number of attempts, success rate on first attempt, view of the vocal cords and perceived subjective safety until successful intubation.

**Results:**

In our study group of 94 participants there was no significant difference in the total time (mean VL: 34 s (±24 s); DL: 37 s (±28 s), *p* = 0.246), while the number of intubation attempts using VL was significantly lower (mean VL: 1.22 (±0.53); DL: 1.37 (±0.60), *p* = 0.023). Success rate of VL during the first attempt was significantly higher (VL: 84%; DL 69%, *p* = 0.016), view of the vocal cords was significantly better and perceived subjective safety was increased using VL.

**Conclusions:**

Our study results suggest that with rather inexperienced operators, VL can be a useful tool to increase rate of successful endotracheal intubation of VLBWI and to improve their perceived safety during the procedure, which may have an impact on mortality and/or morbidity.

## Introduction

Treatment of high-risk preterm infants remains a challenge for all caretakers. Sometimes less experienced pediatricians, anesthesiologists and even obstetricians, midwifes and nurses have to provide respiratory support early after birth, as neonatologists are not readily available in some settings. The tendency to use non-invasive respiratory support in these situations is one reason tracheal intubation is less frequently performed in recent years and therefore individual caretakers' exposure to this procedure is reduced (1).

Although non-invasive respiratory support is often helpful to stabilize high-risk preterm infants immediately after birth, endotracheal intubation may still be needed in many cases (2). In addition to endotracheal intubation for respiratory failure, intratracheal surfactant application *via* LISA (Less Invasive Surfactant Administration) or INSURE (Intubation-Surfactant-Extubation) strategies, requires laryngoscopy to access the trachea.

From the 1970s to the 2000s, technical and medical developments such as the use of continuously positive airway pressure, modern ventilators and the development of surfactant as well as prenatal steroid administration have reduced mortality in premature infants. However, current data show that the incidence of preterm birth remains between 5%–10% in the last 13 years in Europe (3). Mortality rates of neonates and the rates of neurodevelopmental sequelae have plateaued during the last 10–15 years (4, 5). In contrast the general use of video laryngoscopy (VL) has definitely increased over the past 10 years, but the utilization varied widely from NICU to NICU. Current data is sparse but e.g., in the United States the use varied from 3% to 64% in 2015–2017 (6).

Numerous studies in adults have shown that VL is superior to classic laryngoscopes in many situations. VL improved the view of the glottis in both uncomplicated and difficult intubation situations, increased the success rate in the first attempt at intubation, and reduced the rate of complications, such as damage to oral structures, bradycardias and brief hypoxia (7–12). Furthermore, several studies in children have confirmed the advantages of VL in difficult intubation conditions and the possibility to reduce TIAEs (tracheal intubation associated events) (13–15). In particular, VL has shown that it is a suitable and helpful tool for teaching inexperienced medical professionals (13, 16–18).

However, data on the advantages and disadvantages of VL for endotracheal intubation in very premature infants especially by rather inexperienced healthcare providers are not yet sufficient to make definitive recommendations for its general use. Therefore, our aim was to investigate whether video laryngoscopy is a useful tool for improving airway management in very premature infants in a setting of standardized neonatal resuscitation classes. Given the fact that preterm neonates are sometimes very difficult to intubate and extremely prone to hypoxic injury, there is a potential to reduce acute complications during the procedure, and to reduce mortality and long-term morbidity in this population.

## Methods

### Information and consent

Ethical and professional legal assessment of the study concept was carried out by the local ethics committee of the University of Ulm and the study was approved (application number 111/17-FSt/Sta). Before participating in the study, informed written consent was obtained from all study participants.

### Sample size calculation

The study was planned with a randomized cross-over study design [either VL first and direct laryngoscopy (DL) thereafter, or vice-versa]. To show a difference of 5 s in time to successful intubation at a significance level of 5% and a power of 95% a total number of cases of *n* = 29 was calculated (paired *t*-test). However, in case significant carry-over effects would be detected, we also calculated a group size of *n* = 45 subjects, when data obtained from the initial allocation to the two groups would be analyzed by using an unpaired *t*-test. The sample size calculation was carried out using the G * Power program (version 3.1.9.2, Heinrich Heine University Düsseldorf). We then decided to include 90 subjects into the study, 45 in each group.

### Implementation and documentation

Study data collection was performed between 11/2016 and 12/2017. Study participants were physicians, midwives and nurses with predominately little endotracheal intubation experience participating in standardized training courses for neonatal resuscitation according to the Neonatal Resuscitation Program (NRP), endorsed by the American Academy of Pediatrics.

After a standardized briefing of the chapter on endotracheal intubation, the participants were randomly allocated to the groups “video laryngoscopy first” and “direct laryngoscopy first” using sealed envelopes and were crossed over to the alternate allocation thereafter. All participants received a questionnaire to relate to their own level of experience in general endotracheal intubation and some additional parameters.

The intubation manikin used is a realistic simulation manikin of a premature baby of 25 weeks gestational age (“Premature Anne ™”, Laerdal Medical GmbH, Puchheim, Germany). The video laryngoscope (“InfantView” Acutronic Medical Systems, Hirzel, Switzerland, with a Miller blade, size 0) was compared to a standard conventional laryngoscope (Karl Storz SE & Co. KG, Tuttlingen, Germany with a Miller blade, size 0). Orotracheal intubations were performed using an endotracheal tube ID 3.0 (VYGON GmbH & Co. KG, Aachen, Germany) with a stylet after being instructed in a standardized face to face group lesson for endotracheal intubation (PowerPoint® presentation including a video). The total time to successful intubation as the primary outcome was measured by study personnel using a stopwatch and defined as the time from the first insertion of the laryngoscope into the mouth of the manikin until its removal. Success was subsequently checked by the experienced study personnel using VL. If more than one attempt was needed for successful intubation the total time corresponded to the sum of the individual intubation attempts. There was no limitation of time or number of attempts for each participant. In addition, secondary outcomes were the number of intubation attempts needed for successful intubation, the success rate during the first intubation attempt, and the view of the glottis *via* the Percentage of glottis opening (POGO) Score (19) and the perceived safety using a rating system from 1 (maximum safety)–6 (maximum unsafety). The latter 2 variables were rated by the study participants.

### Data analysis

Measured data were initially tabulated into a Microsoft Excel® table. The data were then transferred to SPSS Statistics 24 for Microsoft Windows (Microsoft, Redmond, Washington, United States) and compared independently and depending on the intubation experience.

Outcome variables were tested for normal distribution using a Shapiro–Wilk test. Carry-over effects were checked and could be statistically ruled out due to an appropriate time of at least 3 min between the two intubation procedures. For analysis of the primary and secondary target criteria, the difference in the mean value of the intra-individual differences of the respective sequence group “First direct laryngoscopy” minus the mean value of the sequence group “First video laryngoscopy” divided by 2 was calculated and compared. A *p*-value <0.05 was rated as statistically significant.

Outcome variables were analyzed depending on previous intubation experience in the same way, for which all test subjects were divided into subgroups according to their experience of fewer or equal and more than 10 intubations performed.

Continuous data are reported as mean ± standard deviation for normally distributed data where unpaired *t*-tests or paired *t*-tests were used. For non-parametric distribution data is reported as median (min–max) and Mann–Whitney*U*-tests were used.

## Results

Overall results concerning the primary and secondary target can be seen in Table 1.

**Table 1:** Overview of the primary and secondary study results.

	Video laryngoscopy***	Direct laryngoscopy***	Significance**
**Total time:**			
Independent of intubation experience	33.93 s (24.05)	37.48 s (27.69)	*p* = 0.246
Depending on intubation experience			
≤10 intubations:****	36.50 s (25.67)	40.33 s (28.73)	*p* = 0.311
>10 intubations:*****	26.20 s (16.70)	29.25 s (22.99)	*p* = 0.709
**Number of attempts:**			
Independent of intubation experience	1.22 (0.53)	1.37 (0.60)	*p* = 0.023
	Mdn (Min-Max): 1 (1-3)	Mdn (Min/Max): 1 (1/3)	
Depending on intubation experience			
≤10 intubations:****	1.28 (0.59)	1.42 (0.62)	*p* = 0.077
>10 intubations:*****	1.04 (0.20)	1.20 (0.50)	*p* = 0.052
**Success on first attempt**			
Independent of intubation experience	84%	69%	*p* = 0.016
Depending on intubation experience			
≤10 intubations:****	78%	64%	*p* = 0.061
>10 intubations:*****	95%	83%	*p* = 0.156
POGO* **Score in %**			
Independent of intubation experience	87.8 (12.8)	75.6 (22.1)	*p* < 0.001
Depending on intubation experience			
≤10 intubations:****	86.0% (12.9)	71.5% (21.2)	*p* < 0.001
>10 intubations:*****	92.5% (11.5)	87.5% (21.1)	*p* = 0.152
**Perceived safety in grades:**			
Independent of intubation experience	2.32 (1.04)	2.98 (1.15)	*p* < 0.001
	Mdn (Min/Max): 2 (1/5)	Mdn (Min/Max): 3 (1/6)	
Depending on intubation experience			
≤10 intubations:****	2.42 (1.02)	3.11 (1.16)	*p* < 0.001
>10 intubations:*****	2.04 (1.02)	2.62 (1.09)	*p* = 0.022

* POGO Score: Percentage Of Glottis Opening Score.

** Unpaired *t*-test/Mann–Whitney *U*-test, Chi Quadrat test (Success in first attempt); Calculated with the intra-individual difference of the individual subjects and sequence groups.

*** Mean ± (standard deviation); Calculated with the absolute values of both periods of the individual subjects during video laryngoscopy and direct laryngoscopy.

**** 70 subjects.

***** 24 subjects (17 with 11–100; 7 with >100 intubations).

### Study population

The total study population of 94 participants consisted of 43 physicians, 29 nurses and 22 midwifes. 70 study subjects had experience with ≤10 and 24 subjects had experience with >10 endotracheal intubations. (Consisting of 17 participants with 11–100 intubations and 7 with more than 100 intubations) The general experience in using video laryngoscopy was limited.

### Exclusion of carry-over effects

Carry-over effects could be statistically excluded in the total study population and within the two subgroups with higher or lower previous intubation experience as seen in Table 2.

**Table 2:** Exclusion of carry-over effects of the study results.

	Carry-Over Effect		Unpaired *t*-test/Mann–Whitney *U*-test
**Total time:**			
Independent of intubation experience	Total:	No	*p* = 0.898
Depending on intubation experience	>10 intubations:	No	*p* = 0.104
	≤10 intubations:	No	*p* = 0.219
**Number of attempts:**			
Independent of intubation experience	Total:	No	*p* = 0.986
Depending on intubation experience	>10 intubations:	No	*p* = 0.052
	≤10 intubations	No	*p* = 0.268
POGO* **Score in %**			
Independent of intubation experience	Total:	No	*p* = 0.801
Depending on intubation experience	>10 intubations:	No	*p* = 0.736
	≤10 intubations:	No	*p* = 0.781
**Perceived safety in grades:**			
Independent of intubation experience	Total:	No	*p* = 0.821
Depending on intubation experience	>10 intubations:	No	*p* = 0.716
	≤10 intubations:	No	*p* = 0.706

* Percentage of glottis opening score.

### Total time

**Figure 1:**
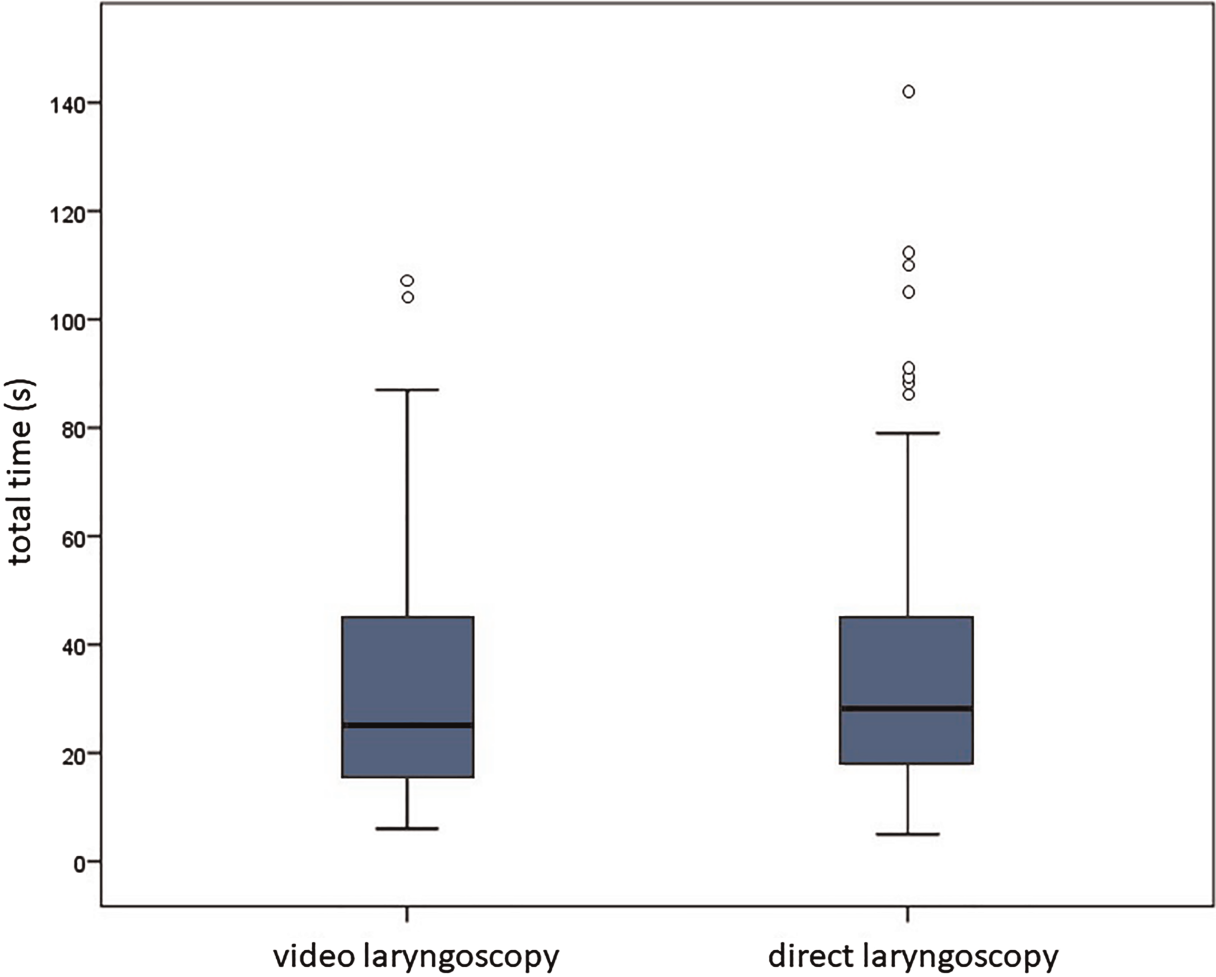
Total time for intubation in seconds (s): video laryngoscopy: median, min/max: 26, 6/107; direct laryngoscopy: median, min/max: 29, 5/142.

For the total time to successful intubation, a mean time of 33.9 s ± 24.1 s was recorded for VL and 37.5 ± 27.7 s for DL (Figure 1). This resulted in a mean difference of 3.6 s for the respective intubation techniques and corresponding subgroups, which was not statistically different (*p* = 0.246). When subgroups were analyzed depending on previous intubation experience (≤10 and >10 intubations), there was no significant difference in total time to successful intubation.

### Number of attempts

However, with VL significantly less intubation attempts were needed as compared with DL (VL: mean: 1.22 attempts, median (min/max): 1 (1/3) vs. DL: mean: 1.37 attempts, median (min/max): 1 (1/3)), resulting in a mean difference of 0.15 attempts (*p* = 0.023). We found trends towards fewer attempts until success favoring VL depending on previous intubation experience in both subgroups.

### First attempt success rate

With VL, 79 intubations (84%) were successful on first attempt and 15 intubations required more than one attempt. With DL 65 intubations (69%) were successful with the first attempt and 29 intubations after more than one attempt (Table 3). This difference was significant favoring VL regardless of previous intubation experience. In subgroup analyses depending on the previous experience there was a trend favoring VL in the group of very low experience (<10 intubations).

**Table 3:** Success rate of the first attempt of intubation.

	Video laryngoscopy	Direct laryngoscopy
Success on first attempt	84%	69%
Absolute risk:	79/94	65/94
Relative risk:	1.215	
Relative risk reduction	17.85%	
Absolute risk reduction	15%	
Number needed to treat	6.66	

### POGO score

The POGO score was significantly higher with the video laryngoscope as compared to the direct laryngoscope: 87.8% ± 12.8% vs. 75.6% ± 22.2%. For very low experienced subjects this difference remained statistically significant.

### Perceived safety

The perceived safety was higher with VL as compared to DL with a mean score of 2.32 points [median (min/max): 2 (1/5)] vs. 2.98 points [median (min/max): 3 (1/6)]. This result was independent of previous intubation experience.

## Discussion

Many studies primarily examine the intubation with VL in term neonates and adults. To our knowledge there is a very limited number of studies concerning that topic with premature infants or manikins simulating very premature infants available in the literature. It should be emphasized, however, that studies with this vulnerable group are particularly important, as the time from alveolar hypoventilation to cerebral hypoxia is considered to be much shorter in this population.

Currently no defined time frame is recommended by the Neonatal Rescuscitation Guidelines for intubation to be safe (20). The mean values of the total time until successful intubation as measured in this study, were 33 and 37 s for the two groups respectively, which is shorter than the intubation time reported in a Cochrane meta-analysis of newborn intubations showing mean values of around 56–60 s (21). Several studies show that adequate oxygenation and ventilation should be achieved within the first minute after birth (22, 23).

Fiadjoe et al. divided the total time to intubation into two phases. The objective of the first phase (time to best view) was to obtain the best view of the glottis whereas the objective of the second phase was to advance the endotracheal tube into the trachea (endotracheal passage time (24). In our study, no precise data were collected regarding the two phases of intubation described above. Nonetheless, it was observed that the difficulties of VL were primarily related to moving the tube within the larynx correctly and to place its tip in the trachea. In contrast, the main problem with DL appeared to be related more to obtain a good view of the glottis. Successful endotracheal advancement of the tube depends on several factors. The use of a stylet and the size and type of blade may play a role. In addition, the specific anatomical characteristics of the upper and lower airway structures of newborns and infants must be considered. The tongue is relatively large in relation to the cavity of the hypopharynx and the maximum opening of the mouth. Additionally the tracheal entry at the glottis is angled in relation to the position of the larynx making intubation with VL difficult (11). Due to these specific preconditions disadvantages using VL can be that there is no direct line of vision and no 3-dimensional vision. Good hand-eye coordination is therefore necessary in order to avoid injuries to the infant during intubation (13).

Fiadjoe et al. hypothesized that intubation experience facilitates indirect intubation technique and therefore the longer time for the endotracheal tube advancement using VL can be compensated (24). In contrast, the evaluation of the total time in this study did not show any significant differences in relation to previous intubation experience. In adults, several studies analyzed total time of intubation using VL in subjects with intubation experience, but results were mixed. Aziz et al. reported a longer intubation time using VL compared to DL (12). In contrast, Jungbauer et al. reported that VL led to a significantly shorter intubation time than DL (9). Malik et al. found comparable overall times to successful intubation using both techniques (25). In summary, the data available on the influence of intubation experience using VL on overall intubation time is very limited and results are heterogeneous. Data is sparse in pediatric, neonatal and especially in premature infants. Currently available data suggest no significant negative or positive influence of VL on the intubation time.

Our study results suggest that VL as compared to DL in the hands of a rather inexperienced healthcare providers would reduce intubation attempts by one every 7 attempts (NNT = 6.66), which can have an influence on the complication rate as described by Park et al. (26). Other studies describe similar numbers of attempts using VL compared to DL in newborns. The authors of the Cochrane meta-analysis from 2018 do not describe any significant difference in the number of intubation attempts in newborns (23). Iacovidou et al. came to a similar result. However, in their study, a larger number of intubation attempts was calculated in comparison, as they already counted an intubation time of more than 30 s and pulling on the manikins upper lip as an unsuccessful attempt (27).

Complication rates seem to be related to the number of intubation attempts which is emphasized by several studies: R. Park et al. analyzed data from the Pediatric Difficult Intubation Registry and demonstrated that the complication rates were linked to the number of attempts regardless of the type of intubation. Each additional attempt doubled the likelihood of complications (26). The importance of the number of previous intubation attempts is also pointed out by Fiadjoe et al. The authors retrospectively examined the complication rate during intubation in children depending on the type of intubation (DL, VL and fiberoptic intubation) in 1,018 difficult intubations. They found an increased risk of complications with more than three preliminary attempts with DL, with a patient weight of less than 10 kg, and a short thyromental distance (28).

Higher success rates of VL have been described in adult studies, as confirmed by Noppens et al. (7), Sakles et al. (8, 29), Jones et al. (10) and Mort et al. (30). The results of the present study also showed a significantly higher success rate of VL on the first intubation attempt compared with DL. This finding is particularly interesting, as total time for intubation tended to be shorter using VL, indicating, that the higher success rate was not achieved by a longer intubation time. We expected that in the subgroup analysis previous intubation experience would be a modifier for this result. However, success rates were no longer significantly different. This may be due to loss of power to show differences in smaller sample sizes. However, if the observed trend towards less attempts needed to successfully intubate using VL would be true for both groups, this would suggest that the use of VL may be beneficial for more experienced health care providers as well.

Other studies in children describe different success rates of VL in the first attempt at intubation compared to DL. Donoghue et al. concluded that VL only increased the success rate of the first attempt at intubation in adults, but not in children and newborns (31). In the meta-analysis of studies in children by Sun et al., VL had a similar success rate in the first attempt comparing with DL in newborns (32). In contrast to the results of Donoghue et al. and Sun et al. the authors of the 2018 Cochrane meta-analysis describe a significantly improved success rate in the first attempt with VL in newborns. The study subjects of the studies were exclusively beginners with little intubation experience (23). Wallenstein et al. pointed out the importance of a short duration of intubation procedures and a low number of intubation attempts in premature infants. They described that intubations, successful in the first attempt, were associated with death or adverse neurodevelopmental outcome in 29% of the cases, as compared to 53% with multiple attempts (33). Their results concur with results of a previous study by O’Donnell et al. (34). It should be noted, however, that association does not imply necessarily causality.

This study showed that VL significantly improved the view of the glottis. It is interesting to note that, depending on the intubation experience, the delta in the recorded POGO scores decreases. The possible cause is that the more experienced staff is also able to obtain a better view of the glottis during DL and the advantages of the video laryngoscope's field-of-view optics are attenuated accordingly. The results obtained in our study, however, are prone to subjective reporting by the intubating person, which is difficult to objectify and may result in bias. An objective assessment of the POGO Score could have been obtained during VL, but not in the comparison group (DL). By using the statistical advantages of the cross-over design and the representative quality of the POGO Score (19) we tried to reduce this bias.

Other studies also support an improved vision using VL. Sun et al. in his meta-analysis on the effectiveness of VL shows that this technique improved the view of the glottis during intubations with normal airways as well as with difficult intubations in children (32). Similar findings were reported by White et al., Fiadjoe et al. and Kim et al., who described a higher POGO score in VL for intubation of newborns and children (24, 35, 36). However, the better view of the glottis does not necessarily guarantee a shorter intubation time in general pediatric intubations, as described by Fiadjoe et al. (24).

Perceived safety during tracheal intubation may be of crucial importance for inexperienced staff members as feeling “unsafe” while performing a potential live-saving procedure may be quite stressful. However, it is a subjective parameter. In our study, perceived safety was better regardless of previous intubation experience with VL. Rabiner et al. came to a similar conclusion (37). Since, in particular less experienced subjects, have difficulties performing an intubation, a better feeling of safety may be helpful in reducing the stress reaction during planned and especially during emergency intubations.

Our experimental setup was standardized during all training classes and the subjects were not instructed during the procedures. Therefore, no statement can be made about improved performance once instructions by an experienced trainer would be provided during intubation. Fiadjoe et al. assessed training and teaching in their study and concluded that VL is a useful educational tool for two main reasons. First, the intubating person can identify the anatomical structures more easily because of the magnifying optics of the laryngoscope and an enlarged field of view is obtained, and second, the more senior supervising person can follow the procedure on the screen, and provide guidance if needed (13, 16). This was confirmed by a similar study by O'Shea at al. who used the video laryngoscope to teach unexperienced caregivers in direct laryngoscopy (16).

Orotracheal intubation is the preferred method in many countries across the world and was performed due to a better feasibility and comparability with the general intubation experience of the study population although nasotracheal intubation is an alternative technique of neonatal intubation in NICUs, commonly used in Europe.

A setting with a premature infant of 25 weeks of gestation was simulated in this study using a state-of-the-art manikin in contrast to similar studies, where manikins were mostly used to represent infants aged 1–9 months or adults (27, 31, 37). However, it is important to consider that a true intubation setting cannot be fully achieved by simulating the procedure in a very premature infant using manikins. First, there is a lack of anatomical variance when using only one single manikin. Second, in a real intubation setting, distinct reduction of the patients' muscular tone as well as aggravating conditions due to saliva, blood or fogging of the optics are to be expected, which can only be simulated with considerable additional effort. However, by using a manikin it is possible to have a better standardization of the experimental setting and the condition of the patient.

The POGO Score was used to document the caregivers' view of the glottis. In contrast to the Cormack Lehane Score, the POGO Score offers a higher consistency and comparability in the evaluation for different intubating subjects (19). Both the POGO score and the assessment of the perceived security are, however, subject to the subjective perception of the caregivers. The score was nonetheless evaluated by the operator himself to make it comparable to the not verifiable score of the direct laryngoscopy. Due to the study design, it was possible to calculate the intraindividual difference so that subjective differences between the operator themselves shouldn’t falsify the total results.

When evaluating similar studies, it should also be noted that video laryngoscopes from different manufacturers and different technologies were used. The Infant View from Acutronic which was used in this study has some similarities compared to the Storz video laryngoscope from Karl Storz (Tuttlingen, Germany) and the Trueview video laryngoscope from Truphatek International Ltd (Netanya, Israel). All three video laryngoscopes have camera optics located on the distal third of the Miller blade. The structure of the blade and the handle are similar to a direct laryngoscope with a straight Miller blade, and DL is also possible with all three video laryngoscopes. O'Shea et al. used this advantage of the Infant View from Acutronic to show that video laryngoscopy is a superior tool in teaching neonatal intubation (16). Other VL systems have a guiding tunnel for the tracheal tube, which is intended to facilitate placement of the tube between the glottis during VL, which may be difficult in real circumstances. However, this means that intubation using DL is no longer possible. Fiadjoe et al. examined various video laryngoscopes for children in a comparative meta-analysis (13). He described that the non-tunneled Storz video laryngoscope in children achieved similar results in total intubation time, field of view and ease of use as compared with the tunneled Glidescope. In a study with adults referenced by Fiadjoe, users of the Storz video laryngoscope performed even better than with the Glidescope. However, the authors point out that the similarity of the Storz video laryngoscope to a classic direct laryngoscope could falsify the results due to previous handling experience which favors the use of a similar devices independent of the video-technique (13).

The definition of subgroups of our study population was based on the reported numbers of intubation experience. However, it is unclear if the arbitrarily chosen cut-off of 10 intubations is enough for labelling “no experience in endotracheal intubation” and “some experience in endotracheal intubation”. However, our participants were rather less experienced, and a higher threshold would have reduced the group size of the subjects with some experience even further. The definition of experience in endotracheal intubation is clearly subjective and depends on factors like talent, stress level, instruments used and patient's individuality. In two studies with adult patients De Oliviera et al. and Konrad et al. described that 40 intubations are necessary to achieve an average success rates of 80% (38, 39).

## Conclusions

The results of our study suggest that using VL can be a useful tool to improve the rate of success and number of attempts when inexperienced healthcare providers have to perform endotracheal intubation in high-risk premature infants which may have an impact on child mortality and/or morbidity. The advantages include the possible use of VL as a teaching tool by guiding less experienced caretakers during the intubation process and to improve their perceived safety during the procedure, which may be an important aspect of staff performance. Further studies with larger study populations of premature infants will be useful to prove whether or not VL can decrease complication rates of endotracheal intubation and thus improve patient safety in situations where an experienced neonatologist is not available.

## Data Availability

The raw data supporting the conclusions of this article will be made available by the authors, without undue reservation.

## References

[1] Leone TA Rich W Finer NN. Neonatal intubation: success of pediatric trainees. J Pediatr. (2005) 146:638–41. 10.1016/j.jpeds.2005.01.029 15870667

[2] Perkins GD Gräsner JT Semeraro F Olasveengen T Soar J Lott C Executive summary: european resuscitation council guidelines 2021. Notfall und Rettungsmedizin. (2021) 24:274–345. 10.1007/s10049-021-00883-z 34093077 PMC8170635

[3] Berger R Abele H Garnier Y Kuon R Rath W Maul H. Premature birth: epidemiology, prediction and prevention. Gynakologe. (2020) 53:331–7. 10.1007/s00129-020-04584-5

[4] Saigal S Doyle LW. An overview of mortality and sequelae of preterm birth from infancy to adulthood. Lancet. (2008) 371:261–9. 10.1016/S0140-6736(08)60136-1 18207020

[5] Handley SC Sun Y Wyckoff MH Lee HC. Outcomes of extremely preterm infants after delivery room cardiopulmonary resuscitation in a population-based cohort. J Perinatol. (2015) 35:379–83. 10.1038/jp.2014.222 25521563 PMC4414658

[6] Grunwell JR Kamat PP Miksa M Krishna A Walson K Simon D Trend and outcomes of video laryngoscope use across PICUs. Pediatr Crit Care Med. (2017) 18:741–9. 10.1097/PCC.0000000000001175 28492404 PMC6317345

[7] Noppens RR Geimer S Eisel N David M Piepho T. Endotracheal intubation using the C-MAC ® video laryngoscope or the macintosh laryngoscope : a prospective, comparative study in the ICU. Crit Care. (2012) 16:R103. 10.1186/cc11384 22695007 PMC3580658

[8] Sakles JC Mosier J Chiu S Cosentino M Kalin L. A comparison of the C-MAC video laryngoscope to the macintosh direct laryngoscope for intubation in the emergency department. YMEM. (2012) 60:739–48. 10.1016/j.annemergmed.2012.03.031 PMC4532403 22560464

[9] Jungbauer A Schumann M Brunkhorst V Börgers A Groeben H. Expected difficult tracheal intubation : a prospective comparison of direct laryngoscopy and video laryngoscopy in 200 patients. Br J Anaesth. (2009) 102:546–50. 10.1093/bja/aep013 19233881

[10] Jones BM Agrawal A Schulte TE. Assessing the efficacy of video versus direct laryngoscopy through retrospective comparison of 436 emergency intubation cases. Japanese Soc Anesthesiol. (2013) 27:927–30. 10.1007/s00540-013-1651-3 23760512

[11] van Zundert AAJ Maassen RLJG Hermans B Lee RA. Videolaryngoscopy - making intubation more successful. Acta Anaesthesiol Belg. (2008) 59:177–8. PMID: 19051449

[12] Aziz M Dillman D Fu R Brambrink A. Comparative effectiveness of the C-MAC video laryngoscope versus direct laryngoscopy in the setting of the predicted difficult airway. Anesthesiology. (2012) 116:629–36. 10.1097/ALN.0b013e318246ea34 22261795

[13] Fiadjoe JE Kovatsis P. Videolaryngoscopes in pediatric anesthesia : what’s new ? MINERVA Anestesiol. (2014) 80:76–82. PMID: 24002465

[14] Grgurich E Arnemann C Amon K Horton R Carlson JN. Just-in-Time video laryngoscopy versus direct laryngoscopy for neonatal intubation. J Perinat Neonatal Nurs. (2016) 30:367–71. 10.1097/JPN.0000000000000216 27776036

[15] Tippmann S Haan M Winter J Mühler AK Schmitz K Schönfeld M Adverse events and unsuccessful intubation attempts are frequent during neonatal nasotracheal intubations. Front Pediatr. (2021) 9:1–7. 10.3389/fped.2021.675238 PMC8144442 34046376

[16] O’Shea JE Thio M Kamlin CO McGrory L Wong C John J Videolaryngoscopy to teach neonatal intubation: a randomized trial. Pediatrics. (2015) 136:912–9. 10.1542/peds.2015-1028 26482669

[17] Low D Healy D Rasburn N. The use of the BERCI DCI Ò video laryngoscope for teaching novices direct laryngoscopy and tracheal intubation*. Anaesthesia. (2008) 63:195–201. 10.1111/j.1365-2044.2007.05323.x 18211452

[18] Moussa A Luangxay Y Tremblay S Lavoie J Aube G Savoie E Videolaryngoscope for teaching neonatal endotracheal intubation: a randomized controlled trial. Pediatrics. (2016) 137:e20152156. 10.1542/peds.2015-2156 26908701

[19] Ochroch EA Hollander JE Kush S Shofer FS. Assessment of laryngeal view: percentage of glottic opening score. Can J Anesth. (1999) 10:987–90. 10.1007/BF03013137 10522589

[20] Madar J Roehr C Ainsworth J Ersda H Morley C Rüdiger M Versorgung und reanimation des neugeborenen nach der geburt. Notfall Rettungsmed. (2021) 24:603–49. 10.1007/s10049-021-00894-w PMC8170636 34093078

[21] Lingappan K Arnold JL Fernandes CJ Pammi M. Videolaryngoscopy versus direct laryngoscopy for tracheal intubation in neonates. Cochrane Database Syst Rev. (2018) 6:1–31. 10.1002/14651858.CD009975.pub3 PMC6513507 29862490

[22] Lane B Finer N Rich W. Duration of intubation attempts during neonatal resuscitation. J Pediatr. (2004) 145:67–70. 10.1016/j.jpeds.2004.03.003 15238909

[23] Kattwinkel J. Textbook of neonatal resuscitation. 6th ed. Dallas, Texas, United States: American Academy of Pediatrics and American Heart Associat (2011). 176.

[24] Fiadjoe JE Gurnaney H Dalesio N Sussman E Zhao H Zhang X A prospective randomized equivalence trial of the GlideScope cobalt® video laryngoscope to traditional direct laryngoscopy in neonates and infants. Anesthesiology. (2012) 116:622–8. 10.1097/ALN.0b013e318246ea4d 22270505

[25] Malik MA Subramaniam R Maharaj CH Harte BH Laffey JG. Randomized controlled trial of the pentax AWS w, glidescope w, and macintosh laryngoscopes in predicted difficult intubation. Br J Anaesth. (2009) 103:761–8. 10.1093/bja/aep266 19783539

[26] Park R Peyton JM Fiadjoe JE Hunyady AI Kimball T Zurakowski D The efficacy of GlideScope ® videolaryngoscopy compared with direct laryngoscopy in children who are difficult to intubate: an analysis from the paediatric difficult intubation registry. Br J Anaesth. (2017) 119:984–92. 10.1093/bja/aex344 29028952

[27] Iacovidou N Bassiakou E Stroumpoulis K Koudouna E Aroni F Papalois A Conventional direct laryngoscopy versus videolaryngoscopy with the GlideScope®: a neonatal manikin study with inexperienced intubators. Am J Perinatol. (2011) 28:201–6. 10.1055/s-0030-1266157 20827657

[28] Fiadjoe J Nishisaki A Jagannathan N Hunyady A Greenberg R Reynolds P. Airway management complications in children with difficult tracheal intubation from the pediatric diffi cult intubation (PeDI) registry: a prospective cohort analysis. Lancet Respir. (2015) 4:1–12. 10.1016/S2213-2600(15)00508-1 26705976

[29] Sakles JC Mosier JM Chiu S Keim SM. Tracheal intubation in the emergency department: a comparison of GlideScope® video laryngoscopy to direct laryngoscopy in 822 intubations. JEM. (2012) 42:400–5. 10.1016/j.jemermed.2011.05.019 21689899

[30] Mort TC Braffett BH. Conventional versus video laryngoscopy for tracheal tube exchange: glottic visualization, success rates, complications, and rescue alternatives in the high-risk difficult airway patient. Anesth Analg. (2015) 121:440–8. 10.1213/ANE.0000000000000825 26111264

[31] Donoghue A Ades A Nishisaki A Deutsch E. Videolaryngoscopy versus direct laryngoscopy in simulated pediatric intubation. Ann Emerg Med. (2013) 61:271–7. 10.1016/j.annemergmed.2012.09.008 23083969

[32] Sun Y Lu Y Huang Y Jiang H. Pediatric video laryngoscope versus direct laryngoscope: a meta-analysis of randomized controlled trials. Paediatr Anaesth. (2014) 24:1056–65. 10.1111/pan.12458 24958249

[33] Wallenstein M Birnie K Arain Y Yang W Yamada N Huffmanm L Failed endotracheal intubation and adverse outcomes among extremely low birth weight infants. J Perinatol. (2015) 36:1–4. 10.1038/jp.2015.158 PMC4731260 26540244

[34] O’Donnell C Kamlin C Davis P Morley C. Endotracheal intubation attempts during neonatal resuscitation: success rates, duration, and adverse effects. Pediatrics. (2006) 117:e16–21. 10.1542/peds.2005-0901 16396845

[35] White M Weale N Nolan J Sale S Bayley G. Comparison of the cobalt glidescope® video laryngoscope with conventional laryngoscopy in simulated Normal and difficult infant airways. Paediatr Anaesth. (2009) 19:1108–12. 10.1111/j.1460-9592.2009.03123.x 19659602

[36] Kim J Na H Bae J Kim D Kim H Kim C Glidescope® video laryngoscope: a randomized clinical trial in 203 paediatric patients. Br J Anaesth. (2008) 101:531–4. 10.1093/bja/aen234 18689807

[37] Rabiner J Auerbach M Avner J Daswani D Khine H. Comparison of glidescope videolaryngoscopy to direct laryngoscopy for intubation of a pediatric simulator by novice physicians. Emerg Med Int. (2013) 2013:1–6. 10.1155/2013/407547 PMC3833063 24288617

[38] De Oliveira Filho GR. The construction of learning curves for basic skills in anesthetic procedures: an application for the cumulative sum method. Anesth Analg. (2002) 95:411–6. 10.1213/00000539-200208000-00033 12145063

[39] Konrad C Schüpfer G Wietlisbach M Gerber H. Learning manual skills in anesthesiology: is there a recommended number of cases for anesthetic procedures? Anesth Analg. (1998) 86:635–9. 10.1097/00000539-199803000-00037 9495429

